# Terpenes-Modified Lipid Nanosystems for Temozolomide, Improving Cytotoxicity against Glioblastoma Human Cancer Cells In Vitro

**DOI:** 10.3390/nano14010055

**Published:** 2023-12-24

**Authors:** Tatiana N. Pashirova, Andrey V. Nemtarev, Daina N. Buzyurova, Zukhra M. Shaihutdinova, Mudaris N. Dimukhametov, Vasily M. Babaev, Alexandra D. Voloshina, Vladimir F. Mironov

**Affiliations:** 1 Arbuzov Institute of Organic and Physical Chemistry, FRC Kazan Scientific Center of RAS, 8 Arbuzov St., 420088 Kazan, Russia; a.nemtarev@mail.ru (A.V.N.); daina.buzyurova@gmail.com (D.N.B.); shajhutdinova.z@mail.ru (Z.M.S.); mudaris@iopc.ru (M.N.D.); babaev@iopc.ru (V.M.B.); sobaka-1968@mail.ru (A.D.V.); mironov@iopc.ru (V.F.M.); 2 Alexander Butlerov Institute of Chemistry, Kazan (Volga Region) Federal University, 18 Kremlevskaya St., 420008 Kazan, Russia

**Keywords:** lipid nanoparticles, terpenes, temozolomide, abietic acid, *Abies sibirica Ledeb*. resin

## Abstract

Currently, increasing the efficiency of glioblastoma treatment is still an unsolved problem. In this study, a combination of promising approaches was proposed: (i) an application of nanotechnology approach to create a new terpene-modified lipid system (7% *w*/*w*), using soybean L-α-phosphatidylcholine, N-carbonyl-methoxypolyethylene glycol-2000)-1,2-distearoyl-sn-glycero-3-phosphoethanolamine for delivery of the chemotherapy drug, temozolomide (TMZ, 1 mg/mL); (ii) use of TMZ associated with natural compounds—terpenes (1% *w*/*w*) abietic acid and *Abies sibirica Ledeb*. resin (*A. sibirica*). Different concentrations and combinations of terpene–lipid systems were employed to treat human cancer cell lines T 98G (glioblastoma), M-Hela (carcinoma of the cervix) and human liver cell lines (Chang liver). The terpene–lipid systems appeared to be unilamellar and of spherical shape under transmission electron microscopy (TEM). The creation of a TMZ-loaded terpene–lipid nanosystem was about 100 nm in diameter with a negative surface charge found by dynamic light scattering. The 74% encapsulation efficiency allowed the release time of TMZ to be prolonged. The modification by terpenes of TMZ-loaded lipid nanoparticles improved by four times the cytotoxicity against human cancer T 98G cells and decreased the cytotoxicity against human normal liver cells. Terpene-modified delivery lipid systems are of potential interest as a combination therapy.

## 1. Introduction

Gliomas (GBM) are common malignant brain tumors. The standard clinical protocol for treatment of GBM alongside surgery [1,2] and radiotherapy is chemotherapy with temozolomide (TMZ) [3]. All attempts to develop more effective therapies have been unsuccessful [4] so far. Therefore, it is urgently necessary to improve and create alternative and effective treatments and new therapeutic strategies [5,6]. The low efficiency of TMZ is due to rapid degradation and lack of target specificity. TMZ is converted to 5-(3-methyltriazen-1-yl) imidazole-4-carboxamide (MTIC) and then to 4-amino-5-imidazole-carboxamide (AIC) and methyldiazonium ion which is responsible for DNA methylation [7]. Different strategies were used to improve the effectiveness of TMZ by imparting specificity and sustainability, for example, the multi-targeted approach [8,9] and incorporation into different drug carriers [10,11,12,13]. Nanodrugs against glioblastoma are more effective due to the simultaneous effect of several mechanisms of antitumor action [14,15,16]. The therapeutic action of treatment may be improved by combining immunotherapy with chemotherapy, radiation therapy and targeted therapy [17,18,19]. The creation of nanosystems with much greater efficiency through smart chemical engineering is currently underway [20,21]. However, the efficacy of TMZ in targeting delivery systems to brain tumors is still far from optimal. Research attention should focus on the functionalization of nanocarriers to more efficiently cross the blood–brain barrier (BBB) or the use of different routes for administration of TMZ. These could increase TMZ concentration in the brain without systemic complications [22,23].

Among the different types of nanoparticles, classical liposomal systems are the most extensive and well-studied lipid delivery systems to date [24,25,26,27]. Liposomes are the most predominantly successful delivery systems for biomedical applications [28,29,30] and are used in clinics [31]. A recent review summarized the therapeutic effect data concerning TMZ via encapsulation in liposomes to create realistic nanomedicines for GBM treatment [32]. It is known that stealth liposomes, namely TMZ-loaded PEGylated liposomes, are able to overcome the disadvantages of classical liposomes [33]. Therefore, N-(carbonyl-methoxypolyethylene glycol-2000)-1,2-distearoyl-sn-glycero-3-phosphoethanolamine, sodium salt) (DSPE-PEG_2000_) was chosen as a PEGylation agent. Previously, we have shown [34] that modification of lipid nanoparticles via DSPE-PEG_2000_ contributes to the vehicle stabilization and improves the therapeutic effect in vivo in rats.

Therefore, to solve the main problems of enhancing and specifically targeting delivery and overcoming the BBB, using TMZ-loaded lipid nanoparticles, we used a combined approach. A combinatorial approach using TMZ is one of the promising pathways in the treatment of glioblastoma [35,36,37], taking into account data of synergistic drug combinations [38]. In particular, bioactive natural molecules are of growing interest either as multitarget chemopreventive agents or using drug delivery systems [39,40,41,42]. Natural compounds hold great promise in chemoprophylaxis to reduce the occurrence of cancer. In addition, the use of drug combinations may induce synergy, decrease toxicity [43,44,45,46,47] and activate immunity [48]. Many natural compounds (e.g., resveratrol, quercetin) are known to work synergistically with TMZ [40,49]. In particular, a synergistic effect was observed for a combination of TMZ with cedrol in a GBM animal model [50,51] and the co-encapsulation of quercetin and TMZ into stealth lipid systems [52].

Thus, one of their features is that the natural terpenes can provide and enhance the targeted cancer cell delivery properties [53,54]. In particular, *A. sibirica* resin shows cytotoxic activity against tumor cell lines A549, COLO-205, QGY-7703 and THP-1 [55,56]. An alternative feature is that terpene-containing liposomes called “invasomes” or “terpesomes” with ultra-deformable features can improve penetration enhancement and mucoadhesive properties [57,58]. Terpene-containing lipid nanosystems are of potential interest for targeting the brain and permeating the BBB [59]. *A. sibirica*-modified lipid nanoparticles are able to bypass the BBB via the non-invasive intranasal route [60]. Therefore, different routes for the delivery of TMZ to the CNS, using terpene-modified liposomes can be considered, thereby avoiding interference with first-pass metabolism [61,62]. In addition, inhibition of the inflammatory response and reduction in the risk of tumor recurrence can be achieved through adjuvant cancer therapy with abietic acid [63,64].

Our main goal was to establish targeted green and non-toxic TMZ-loaded PEGylated lipid delivery systems based on soybean L-α-phosphatidylcholine and containing 1% *w*/*w* terpenes *A. sibirica* resin and abietic acid. The combination of TMZ with abietic acid by applying invasomal nanocarriers is one of the first steps in improving the efficiency of TMZ against glioblastoma human cancer cells in vitro.

## 2. Materials and Methods

### 2.1. Chemicals

N-(carbonyl-methoxypolyethylene glycol-2000)-1,2-distearoyl-sn-glycero-3-phosphoethanolamine, sodium salt) (DSPE-PEG_2000_, Avanti polar lipids), L-α-phosphatidylcholine (PC, Soy, 95%, Avanti polar lipids, Alabaster, AL, USA), cholesterol (Ch, Sigma Grade, ≥99%), abietic acid (Aldrich, Saint Louis, MO, USA), temozolomide (TMZ, Sigma, Saint Louis, MO, USA, ≥98%), abietic acid (Sigma, ~75%), *Abies sibirica Ledeb.* resin (*Ab. Sibirica*, Altaivita, 99%).

### 2.2. GC/MS Analysis of A. sibirica Resin

GC/MS analysis was performed on a GC-MS-QP2010 Ultra gas chromatograph–mass spectrometer (Shimadzu, Kyoto, Japan) with a Rtx^®^-5 capillary column (Restek, Bellefonte, PA, USA). Ionization was carried out, using the electron impact (EI) mode at 70 eV. Chromatogram peaks were identified based on the chemical library search, using GC-MS Solution real time analysis, as well as a comparison of linear retention indices and mass spectra with the literature data.

#### 2.2.1. Method A

Capillary column (30 m × 0.32 mm × 0.25 µm film thickness). The injector and ion source temperature were 250 and 210 °C, respectively. The pressure of helium carrier gas was kept at 37.5 kPa with split ratio of 1:100. Sample of 1 µL (*A. sibirica* resin diluted in benzene: ethanol 1:1 (*v*/*v*)) was injected. The initial column temperature was 70 °C and held 2 min; ramped up to 150 °C at the rate of 10 °C/min. Total analysis time was 10 min. MS data were acquired in full scan mode from *m/z* 45–500.

#### 2.2.2. Method B

Capillary column (30 m × 0.32 mm × 0.25 µm film thickness). The injector and ion source temperature were 300 °C. The pressure of helium carrier gas was kept at 34.8 kPa with split ratio of 1:2. Sample of 1 µL (*A. sibirica* resin treated with a silylation derivatization reagent (pyridine:hexamethyldisilazane:chlorotrimethylsilane 5:4:1 (*v*/*v*)) at 100 °C) was injected. The initial column temperature was 60 °C and held 3 min, ramped up to 300 °C at the rate of 3 °C/min and kept at this temperature for 30 min. Total analysis time was 113 min. MS data were acquired in full scan mode from *m*/*z* 35–1090.

### 2.3. Preparation of A. sibirica Resin-, Abietic Acid- and TMZ–Loaded Lipid Nanosystems

PC (5.4% *w*/*w*), Chol, (0.3% *w*/*w*), DSPE-PEG2000 (0.2% *w*/*w*) and *A. sibirica* resin or abietic acid (1% *w*/*w*) and TMZ (0.1% *w*/*w*) were dissolved in ethanol: chloroform (1:1) solvent. The homogeneous solution was kept in a water bath at 60 °C until solvent evaporation to obtain a thin film. Ultra-purified water (Milli-Q, Direct-Q5 UV, Millipore SAS, Molsheim, France) was pre-heated to 60 °C and added to rehydrate the lipidic film at 60 °C to obtain a final lipid concentration of 7% *w*/*w* in Milli-Q water. The solution was stirred under magnetic stirring (750 rpm) (IKA, Koningswinter Germany) for 30 min at the same temperature. Then, the solution was kept for 1.5 h in water bath at 37 °C. The multilamellar liposome solution was extruded 10 times by passage through a polycarbonate membrane of 100 nm pore size (Mini-Extruder Extrusion Technique, Avanti Polar Lipids, Inc., Alabaster, AL, USA).

### 2.4. Characterization of A. sibirica Resin-, Abietic Acid and TMZ-Loaded Lipid Nanosystems

All characteristics (size, polydispersity index and zeta potential) were determined by dynamic light scattering (DLS) measurements, using the Malvern Instrument Zetasizer Nano (Worcestershire, UK). The measured autocorrelation functions were analyzed by Malvern DTS software 7.13, applying the second-order cumulant expansion methods. The size (hydrodynamic diameter) was calculated according to the Einstein–Stokes relationship *D = k_B_T/3πηx*, in which *D* is the diffusion coefficient, *k_B_* the Boltzmann’s constant, *T* is the absolute temperature, *η* is the viscosity, and *x* is the hydrodynamic diameter of nanoparticles. The diffusion coefficient was measured at least in triplicate for each sample. The average error of measurements was approximately 4%. All samples were diluted with ultra-purified water to a suitable concentration and analyzed in triplicate.

Transmission electron microscopy (TEM) was used to monitor the size and the morphology of TMZ-loaded terpene-modified lipid systems. TEM images were obtained, using a Hitachi HT7700 (Tokyo, Japan)Exalens microscope, Japan, camera XR81-DIR (3296 × 2464 pixel). The images were acquired at an accelerating voltage of 100.0 kV. Samples (C = 0.7 μg/mL) were added to a 300 mesh copper grids with continuous carbon formvar support films and dried at room temperature.

#### 2.4.1. IR Spectroscopy

IR spectra of ethanol, ethanol solutions of PC and its mixture with abietic acid and TMZ were recorded, using a Bruker Vector-27 FTIR spectrometer in the 400–4000 cm^−1^ range (optical resolution 4 cm^−1^). The neat PC sample as well as solutions were prepared between CaF_2_ plates. The ethanol solutions were prepared in concentrations: PC 10% *w*/*w*, PC:abietic acid = 1:1.

#### 2.4.2. Encapsulation Efficiency and Loading Capacity

Encapsulation efficiency (*EE*, %) and loading capacity (*LC*, %) were assessed for samples *A. sibirica* resin-, abietic acid- and TMZ-loaded lipid nanosystems. These parameters were determined indirectly by filtration/centrifugation, measuring free TMZ (non-encapsulated) by spectrophotometry. A volume of 0.1 mL of each *A. sibirica* resin-, abietic acid- and TMZ-loaded liposomes was placed in centrifugal filter devices in a Nanosep centrifugal device 3K Omega (Pall Corporation, New York, USA) to separate the lipid and aqueous phase and centrifuged at 10,000 rpm, for 15 min (Eppendorf AG, Hamburg, Germany). Free TMZ was quantified by measuring the absorbance dilution (Figure S1, Supplementary Materials) using a UV-1800 Shimadzu spectrophotometer at 330 nm (the molar extinction coefficient of TMZ at 330 nm is 9525 M^−1^·cm^−1^ at pH = 4 in an acetate buffer after suitable dilution (Figure S1, Supplementary Materials). The encapsulation parameters were calculated against appropriate calibration curve, using the following equations:

(1) $$EE\left(\%\right)=\frac{Total\;amount\;of\;drug-Free\;drug}{Total\;amount\;of\;phospholipid}\times 100\%,$$

(2) $$LC\left(\%\right)=\frac{Total\;amount\;of\;drug-Free\;drug}{Total\;amount\;of\;phospholipid}\times 100\%,$$

#### 2.4.3. In Vitro Drug Release Profile

Samples containing TMZ were analyzed by determining the absorbance at 330 nm for TMZ using a UV-1800 Shimadzu spectrophotometer and HPLC. The extinction coefficients of TMZ in acetate buffer 10 mM, pH = 4, at 330 nm is 9525 M^–1^·cm^–1^ (Figure S1 in Supplementary Materials). All samples were analyzed in triplicate. The HPLC system Agilent 1200 (USA) consisted of an auto sampler; column oven and UV detector were used. Analyses were performed on a C18 column (Zorbax) with dimensions 15 cm × 4.6 mm × 5 mm at 25 °C. The mobile phase was 0.1% trifluoroacetic acid and acetonitrile (90:10, *v*/*v*) and the flow rate was set at 1 mL/min, the injection volume was 10 μL. TMZ expressed maximum absorbance at 326 nm (retention time 2.8 min) while its metabolite (AIC) showed maximum absorbance at 272 nm (retention time 1.7 min). The calibrate curve is presented in Figure S2 (in Supplementary Materials).

### 2.5. Cytotoxicity on Human Cell Lines

The lipid systems were evaluated for their cytotoxic effects against human cancer cell lines T 98G (glioblastoma) and M-Hela (carcinoma of the cervix) and human liver cell lines (Chang liver). M-Hela was obtained from the Collection of the Institute of Cytology of the Russian Academy of Science (St-Petersburg, Russia). Chang liver was acquired from the Collection of the Research Institute of Virology RAS. Cells were cultured in a standard Eagle’s nutrient medium manufactured at Chumakov Institute of Poliomyelitis and Virus Encephalitis (PanEco company, Moscow, Russia) and supplemented with 10% fetal bovine serum and 1% nonessential amino acids. Cytotoxic activity was determined according to the previously described method by counting viable cells, using a multifunctional system Cytell Cell Imaging (GE Healthcare Life Science, Uppsala Sweden) and the application, Cell Viability BioApp [65].

#### Statistical Analysis

IC_50_ was calculated using an online tool: MLA—“Quest Graph™ IC_50_ Calculator.” (AAT Bioquest, Inc., Pleasanton, CA, USA). Cytometric results were analyzed by the Cytell Cell Imaging multifunctional system using the Cell Viability BioApp. The data in the tables and graphs are given as the mean ± standard error.

## 3. Results

### 3.1. Preparation and Characteristics of Terpene-Containing and TMZ-Loaded Lipid Nanosystems

**Figure 1:**
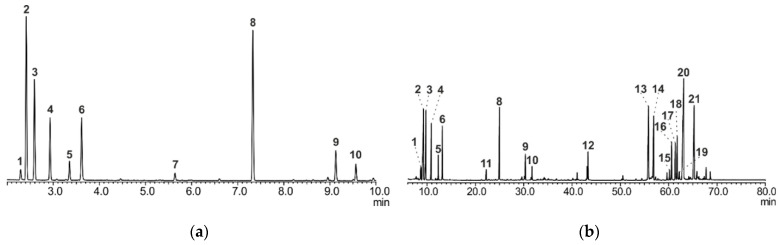
GC-MS chromatograms of *Abies sibirica* resin for identification of components using Methods A (**a**) and B (**b**).

The components of plant material (*A. sibirica* resin) were studied by gas chromatography–mass spectrometry. *A. sibirica* resin was dissolved in the mixture of benzene and ethanol (1:1, *v*/*v*). Volatile components were determined by direct gas chromatographic separation (Method A) and a comparison of linear retention indices with the literature data and NIST mass spectral library data. Low-volatile resin components were determined by gas chromatography after preliminary derivatization with silylation reagents (Method B) in order to increase their volatility. GC-MS chromatograms of *A. sibirica* are presented in Figure 1.

The main components of *A. sibirica* resin were identified (Table S1, Supplementary Materials). Quantitative analysis was performed. The main component of *A. sibirica* resin is abietic acid. The content of abietic acid was about 20%.

All lipid systems were prepared by a simple thin-film hydration method used for classical liposomes [66]. TMZ-loaded terpene-modified lipid nanosystems were characterized by DLS and TEM methods. The characteristics of all lipid formulations are presented in Table 1.

**Figure 2:**
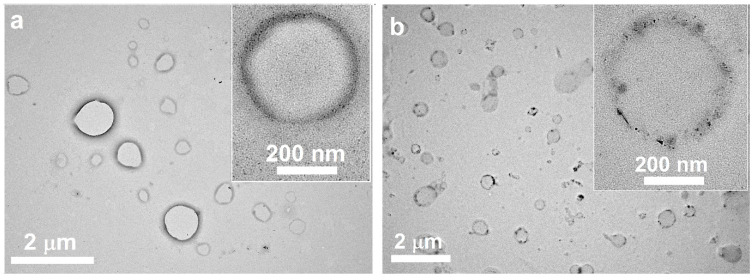
TEM imaging of TMZ-loaded abietic acid (**a**) and *A. sibirica* (**b**) PC/DSPE–PEG_2000_/Ch lipid nanosystems, C_PC_ = 0.58 μg/mL, mQ water, 25 °C. Scale bar is 2 μm and 200 nm are in insert (**a**) and (**b**), respectively.

The size of all lipid systems is about 100 nm. PDI values do not exceed 0.2. The size of lipid nanosystems slightly decreased via modification of terpenes. Zeta potential value is –27 mV in the absence and presence of *A. sibirica*. In the case of abietic acid as terpene, the zeta potential is shifted from –27 mV to –15 mV. The loading of TMZ does not change the characteristics of lipid systems. Despite the fact that the main component of the resin is abietic acid, there are several differences in characteristics of terpene lipid formulations. Abietic acid-modified lipid systems are considered to have a more neutral zeta potential. The differences in values of zeta potential between liposomes may reflect differences in integration into the phospholipid bilayer between *A. sibirica* resin and abietic acid. In the case of TMZ loading, the size of lipid nanosystems slightly increased, and the zeta potential became more negative. The size and zeta potential of the TMZ-loaded abietic acid-modified lipid system are close to the characteristics of the abietic acid-modified lipid system. Freshly prepared samples were used for monitoring the morphology of TMZ-loaded lipid systems. The TEM images are presented in Figure 2a,b, Figures S3 and S4 (Supplementary Materials). As we see, sizes are close to the DLS characteristics. Both lipid systems have a spherical morphology. However, there is a significant difference in the structure of the lipid membrane. Lipid systems containing abietic acid have a dense membrane and an empty core. For the *A. sibirica*-containing systems, the shell has an uneven contrast and the core of the nanoparticles is darker compared to the abietic acid lipid system. In addition, smaller particles are observed. This indicates a multicomponent composition of *A. sibirica*. It is likely that some components of *A. sibirica* incorporate inside lipid nanoparticles. These TEM data confirm that differences in zeta-potential reflect differences in integrating into the phospholipid bilayer between *A. sibirica* resin and abietic acid.

**Figure 3:**
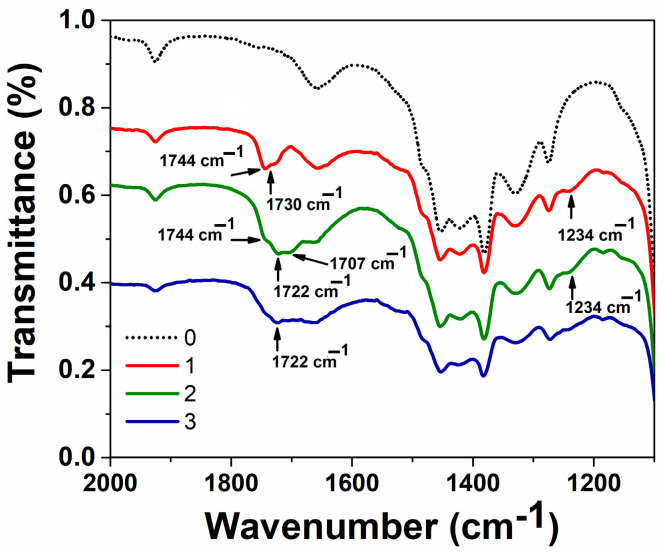
FTIR spectra of PC in ethanol (1, red), mixture of Ph and abietic acid in ethanol (2, olive), mixture of Ph and abietic acid and TMZ in ethanol (3, Royal blue) and ethanol (black).

To study the integration into phospholipid bilayer of abietic acid and possible interactions between PC and abietic acid, we used IR spectroscopy. For this purpose, the infrared spectra of PC (1), the mixture of PC and abietic acid (2) and the mixture of PC, abietic acid and TMZ were recorded (Figure 3). A previous work [67] showed the presence of intermolecular interaction between PC and ethanol: thus, in the IR spectrum of PC, the PO stretching band is shifted to 1234 cm^−1^ compared to the one in the IR spectrum of PC in solid form (1240 cm^−1^), the C=O stretching band appeared at 1735 cm^−1^ in solid form. In ethanol, it was split into the main band at 1744 cm^−1^ and shoulder at 1730 cm^−1^. In the presence of abietic acid (line 2), the C=O stretching band is shifted to 1722 cm^−1^. This indicates the possibility of interactions with the carbonyl groups. The addition of TMZ to the mixture of PC and abietic acid showed a change at the same band and a shift from 1744 cm^−1^ to 1722 cm^−1^ (line 3). This confirms the interaction between polar groups of PC and abietic acid through carbonyl groups.

**Figure 4:**
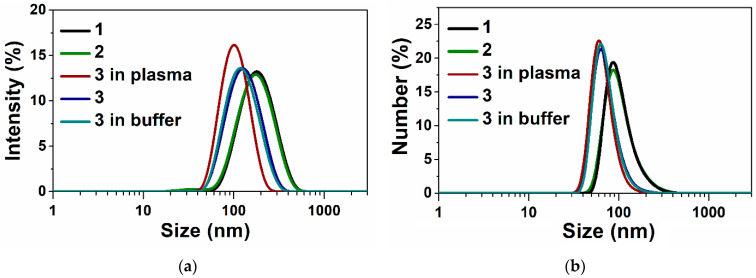
Size distribution using intensity (**a**) and number (**b**) parameters of TMZ-loaded lipid nanosystems (1), *A. sibiric*a–lipid nanosystems (2) and TMZ-loaded abietic acid–lipid nanosystems (3), and monitoring the stability in vitro conditions in Tris buffer (10 mM, pH = 7.4) within 24 h and human plasma within 2 h at 37 °C.

The stability of the TMZ-loaded terpene-modified lipid system was monitored in storage for 4 months in a fridge using a tightly closed glass container in the absence of light at 4 °C (Table 1) and in in vitro conditions in Tris buffer (10 mM, pH = 7.4) within 24 h and human plasma within 2 h at 37 °C (Figure 4). The TMZ-loaded abietic acid-modified lipid system showed good stability. The size and zeta potential were slightly changed.

The encapsulation efficiency and loading capacity of TMZ were calculated using Equations (1) and (2) and are presented in Table 1. It is interesting that despite the different membrane structure, the encapsulation efficiency is the same for both systems.

**Figure 5:**
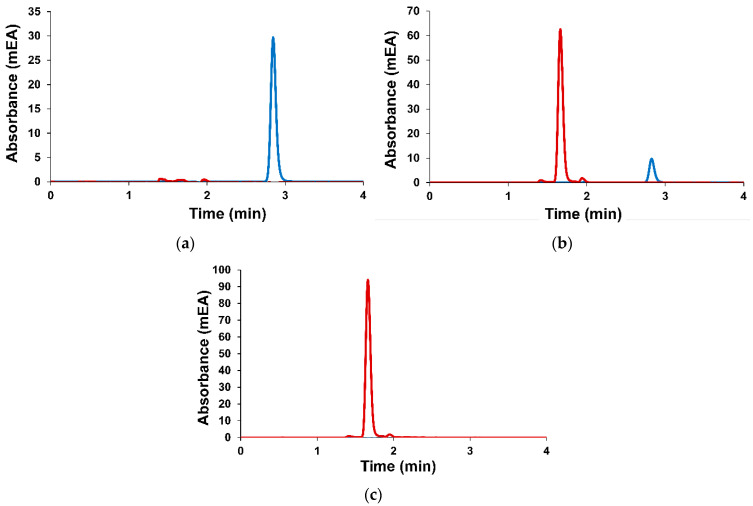
HPLC chromatogram showing TMZ peak at 326 nm (blue) and metabolite peak at 272 nm (red) after (**a**) initial; (**b**) 12 h; (**c**) 24 h in phosphate buffer (0.025 M) at pH = 7.4.

Monitoring of TMZ in phosphate buffer at pH = 7 during 24 h indicates that TMZ is not stable. A decrease in the temozolomide signal and product formation during time at pH = 7 is observed on the HPLC chromatogram (Figure 5).

**Figure 6:**
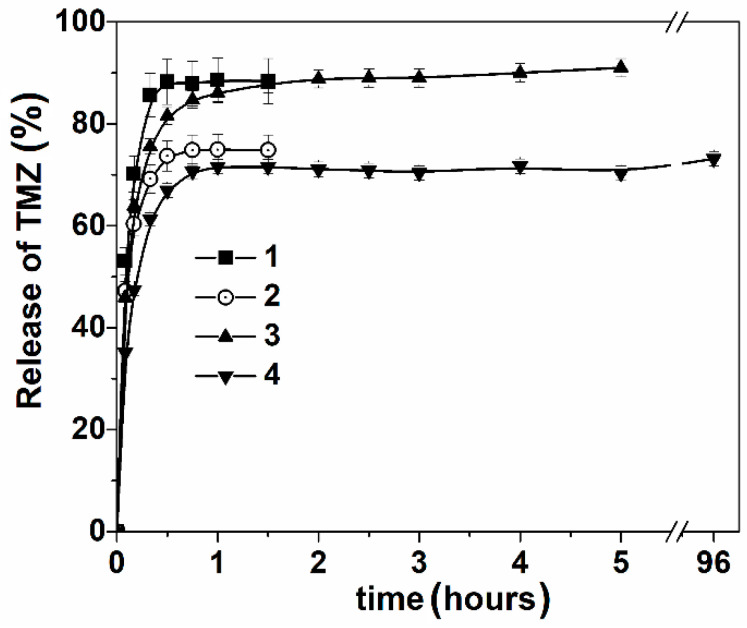
In vitro TMZ release, free solution (1), lipid nanosystems (2), abietic acid–lipid nanosystems (3), *A. sibirica*–lipid nanosystems (4) using the dialysis bag method (n = 3, experiments were replicated in triplicate), acetate buffer (0.01 M), pH = 4, 37 °C.

The release of TMZ was investigated in acetate buffer pH = 4 by spectrophotometry (Figure 6) and HPLC (Figure S5) methods. Absorbance spectra of TMZ in vitro conditions (acetate buffer, pH = 4) show a maximum at λ = 330 nm (Figures S7–S9). An explosion release is observed for free TMZ. Delayed release occurs when lipid delivery systems are used. The slowest release (more than 5 h) is observed for the *A. sibirica*-containing lipid system.

### 3.2. Cytotoxicity on Human Cell Lines

One of the purposes of using the nanotechnological approach is to deliver anticancer agents to tumor cells directly and minimize the cytotoxic effect on healthy tissue and normal cellular function. It is known that abietic acid improves the efficacy of Taxol and shows an antitumor activity on melanoma cells [68]. The effective concentrations, IC_50_, against human cancer T 98G, M-Hela cells and normal Chang liver cells are presented in Table 2.

Abietic acid– and *A. sibirica*–lipid systems show almost the same cytotoxic effect against human cancer cells. However, the *A. sibirica*-containing lipid system is significantly less toxic against Chang liver cells. The ability to induce apoptosis in the M-Hela tumor cell line was studied using the Cytell Cell Imaging multi-functional system and Apoptosis BioApp application. *A. sibirica*– and abietic acid–lipid systems induce apoptosis at concentrations corresponding to the IC_50_ value of these systems. TMZ-loaded–lipid system exhibits a lower toxic effect against cancer and normal cells than free TMZ. But the TMZ-loaded–lipid system modified by terpenes enhance the cytotoxic effect by four times against T 98G cells. At the same time, TMZ-loaded– and terpene-containing–lipid systems were found to be less toxic against normal cells. 

## 4. Discussion

Different approaches and technologies ensure the safe and effective targeted drug delivery to the central nervous system [69]. Permeation enhancers can induce structural changes, affect permeability, open tight contacts of the epithelial layer, and change the fluidity of membranes [70]. Terpenes are involved as permeation enhancers for drugs and provide fluidity, flexibility and organization of the phospholipid membrane [71,72]. Improving the nasal, oral and gastrointestinal bioavailability of drugs, accelerating the opening of the BBB, transdermal, transcorneal, and blood optic nerve barrier and improving the distribution of drugs in brain tissue by a simple bicyclic monoterpene (borneol) was demonstrated [73,74,75]. Improving drug delivery to the brain with borneol-modified nanoparticles via intranasal administration is also known [74,76].

In our work, abietic acid and the multicomponent *A. sibirica* resin were used as terpenes. The use of a multicomponent *A. sibirica* resin in drug delivery systems assumes the exclusion of the extracting process of a single component (abietic acid) from *A. sibirica* resin to also preserve and enhance the beneficial biological properties. Designed abietic acid-containing lipid nanosystems are hypothesized to improve the stability and bioavailability of TMZ.

All components of lipid systems have been carefully selected. Cholesterol is anchored in the phospholipid bilayer and causes the greatest “rigidifying” effect [77]. A hydrophilic polymer such as PEG was used to coat the lipid terpene particles, which inhibits protein adsorption present in the blood plasma, so that the particles can remain masked or invisible to phagocytic cells [78,79,80]. PEGylated lipid nanosytems were prepared by simply mixing with a commercially available PEG-conjugated lipid N-carbonyl-methoxypolyethylene glycol-2000)-1,2-distearoyl-sn-glycero-3-phosphoethanolamine (10% mol) and PC during liposome preparation. All these components provide stability to lipid systems over time (4 months) and in biological fluids (Figure 4). This is not surprising; all lipid nanoparticles have a negative charge, which prevents their aggregation due to electrostatic repulsion. The charge of lipid systems in the absence of PEG is close to neutral [67].

According to the literature data, the polar carboxyl group of abietic acid prevents its location in the terminal methyl group of fatty acyl chains of phospholipids. There is a tendency for it to be located near the polar group of phospholipids, i.e., near the interface with water, where it can form hydrogen bonds with water, as well as establishing other types of interactions with the polar part of phospholipids, disrupting the packing of lipids and lowering their phase transition temperature [81]. Using IR spectroscopy, we found interactions at the carbonyl group (Figure 3). These suggest that molecules of abietic acid intercalate between PC. A change in zeta potential from −27 mV in the case of PC/DSPE–PEG_2000_/Ch to −17 mV for abietic acid-modified PC/DSPE–PEG_2000_/Ch also indicates these interactions. The temozolomide molecule participates in carbonyl interactions (Figure 3, blue line 3) and does not affect the characteristics of lipid systems (Table 1).

TEM images (Figure 2a) also confirm our assumptions due to the presence of dark contrast in the liposome membrane. The structure of abietic acid-modified liposomes is unilamellar and is similar to the described structure of invasomes [57]. The best colloid stability in long term storage (4 months) is observed for TMZ–abietic acid-modified liposomes. The slight change in size and zeta potential compared to the unmodified TMZ-loaded lipid system and TMZ–*Abies sibirica*–lipid systems is primarily due to membrane depolarization and intercalation of abietic acid into the liposome membrane on specific binding sites. This is as for the interaction of alpha-tocopherol with liposomes [82].

In the case of using a multicomponent *A. sibirica* resin, the formation of domains is observed, and an uneven contrast on the membrane of the lipid system (Figure 2b) where the concentration of abietic acid molecules can particularly be high. At the same time, more hydrophilic components of *A. sibirica* could be encapsulated inside (hydrophilic core), which likely affects the zeta potential of the *A. sibirica*-modified PC/DSPE–PEG_2000_/Ch systems (Table 1) and its less toxic properties towards normal human Chang liver cells (Table 2).

Thus, the improvement in the cytotoxicity of temozolomide loaded in abietic acid-modified lipid systems is due to its stability in in vitro conditions at pH = 7.4 and in human plasma in an acidic environment (pH = 4) and via inducing apoptosis by abietic acid–lipid systems. There remains a need to identify mechanisms to highlight the anticancer activity of abietic acid–lipid systems containing temozolomide. Abietic acid definitely inhibits the growth of cancer cells and modulates the permeability of a cell membrane [83,84]. The apoptotic process involves the arrest of the B16-F10 cell cycle, blocking the G0/G1 phase [85], triggering growth arrest of both G2/M cells and the subG0-G1 subpopulation. It also induces overexpression of key apoptotic genes (Fas, FasL, Casp3, Casp8, Cyt-C and Bax) and the downregulation of both proliferation (VEGF, IGFR1, TGF-b) and oncogenic (C-myc and NF-kB) genes [86]. Abietic acid inhibits tumor necrosis factor-α-induced phosphorylation of IκB kinase (IKKα/β) (Ser176/180) and IkBα (Ser32), inhibits also the nuclear translocation of nuclear factor-κB [64]. In addition, abietic acid acts as an adjuvant [87], showing synergistic effect in combination with different chemotherapeutic agents: taxol [68], cisplatin, paclitaxel, gemcitabine, and gefitinib, inducing ferroptosis via the activation of the HO-1 pathway [88]. 

## 5. Conclusions

In conclusion, we designed and characterized *A. sibirica* resin- and abietic acid-modified lipid nanosystems for improving the bioavailability, sustainability, intra-cellular delivery and cytotoxicity effect of temozolomide. Terpene-modified–lipid nanosystems have the ability to interact with free radicals and showed a cytotoxicity effect against human glioblastoma cancer cells with lower toxicity to normal human Chang liver cells and with apoptosis in M-Hela culture cells at IC_50_ concentration. The ability of *A. sibirica*–lipid systems to target the brain tissue is promising for the implementation of terpene-modified–lipid systems combined with anti-cancer drug temozolomide using the nose–brain pathway.

## Figures and Tables

**Table 1:** Size (hydrodynamic diameter, using number distribution and Z-average, nm), polydispersity index (PDI) and zeta potential (ζ, mV) of lipid nanosystems, C_TMZ_ = 0.001% (*w*/*w*), 25 °C.

№	Lipid System	Terpene, % *w*/*w*	Loaded Drug	Size, (nm)	Z_aver_ (nm)	PDI	ζ (mV)	EE (%)	LC (%)
1	PC/DSPE–PEG_2000_/Ch	–	-	78 ± 2	145 ± 1	0.17 ± 0.02	–27.5 ± 0.5	–	–
2	PC/DSPE–PEG_2000_/Ch	*A. sibirica*, 1	-	68 ± 1	137 ± 1	0.2 ± 0.01	–27 ± 1	–	–
3	PC/DSPE–PEG_2000_/Ch	Ab. acid, 1	-	68 ± 1	123 ± 1	0.14 ± 0.01	–15 ± 1	–	–
4	PC/DSPE–PEG_2000_/Ch	–	TMZ	91 ± 1	157 ± 1	0.19 ± 0.01	–32 ± 1	83 ± 1	1.5 ± 0.1
4 *	PC/DSPE–PEG_2000_/Ch	–	TMZ	190 ± 2	297 ± 5	0.16 ± 0.02	–28 ± 1	-	-
5	PC/DSPE–PEG_2000_/Ch	*A. sibirica*, 1	TMZ	91 ± 1	145 ± 1	0.21 ± 0.01	–25 ± 1	74 ± 10	1.4 ± 0.1
5 *	PC/DSPE–PEG_2000_/Ch	*A. sibirica*, 1	TMZ	164 ± 8	225 ± 1	0.21 ± 0.01	–17 ± 1	-	-
6	PC/DSPE–PEG_2000_/Ch	Ab. acid, 1	TMZ	68 ± 1	118 ± 1	0.14 ± 0.01	–18 ± 1	74 ± 15	1.4 ± 0.1
6 *	PC/DSPE–PEG_2000_/Ch	Ab. acid, 1	TMZ	68 ± 1	130 ± 1	0.16 ± 0.01	–15 ± 1	-	-

* 4 month storage.

**Table 2:** IC_50_ values (mg/mL) of the different systems against cells are presented as mean ± standard deviation, after 24 h incubation.

Systems	IC_50_ (mg/mL)			
	T 98G	M-Hela	Chang Liver	M-Hela
Empty lipid system	18.4	15.1	13.5	
Abietic acid–lipid system	0.7	0.98	0.6	45.8 ± 3.5
*A. sibirica–*lipid system	0.75	1.3	1.2	13 ± 1
TMZ	0.04	0.007	0.03	–
TMZ–lipid system	0.3	0.15	0.15	–
TMZ–abietic acid–lipid system	0.07	0.13	0.09	–

## Data Availability

The data presented in this study are available on request from the corresponding author.

## Supplementary Materials

The following supporting information can be downloaded at: https://www.mdpi.com/article/10.3390/nano14010055/s1, Table S1 include identified components of *Abies sibirica* resin; Figures S1–S9 include UV spectra TMZ in acetate buffer, TEM imaging, in vitro TMZ release monitoring by HPLC and UV spectra of TMZ during release process from lipid systems.

## References

[1] Mrugala M.M., Chamberlain M.C., Hutchinson F. (2008). Mechanisms of disease: Temozolomide and glioblastoma—Look to the future. Nat. Clin. Pract. Oncol..

[2] Kim H.J., Kim D.-Y. (2020). Present and Future of Anti-Glioblastoma Therapies: A Deep Look into Molecular Dependencies/Features. Molecules.

[3] Wick W., Weller M., Van Den Bent M., Sanson M., Weiler M., Von Deimling A., Plass C., Hegi M., Platten M., Reifenberger G. (2014). MGMT testing—The challenges for biomarker-based glioma treatment. Nat. Rev. Neurol..

[4] Wen P.Y., Reardon D.A. (2016). Neuro-oncology in 2015: Progress in glioma diagnosis, classification and treatment. Nat. Rev. Neurol..

[5] Stupp R., Lukas R.V., Hegi M.E. (2019). Improving survival in molecularly selected glioblastoma. Lancet.

[6] Rong L., Li N., Zhang Z. (2022). Emerging therapies for glioblastoma: Current state and future directions. J. Exp. Clin. Cancer Res..

[7] Zhang J., Stevens M.F.G., Bradshaw T.D. (2012). Temozolomide: Mechanisms of Action, Repair and Resistance. Curr. Mol. Pharmacol..

[8] Sestito S., Runfola M., Tonelli M., Chiellini G., Rapposelli S. (2018). New Multitarget Approaches in the War Against Glioblastoma: A Mini-Perspective. Front. Pharmacol..

[9] Bahadur S., Sahu A.K., Baghel P., Saha S. (2019). Current promising treatment strategy for glioblastoma multiform: A review. Oncol. Rev..

[10] Fang C., Wang K., Stephen Z.R., Mu Q., Kievit F.M., Chiu D.T., Press O.W., Zhang M. (2015). Temozolomide Nanoparticles for Targeted Glioblastoma Therapy. ACS Appl. Mater. Interfaces.

[11] Afzalipour R., Khoei S., Khoee S., Shirvalilou S., Jamali Raoufi N., Motevalian M., Karimi M.R. (2019). Dual-Targeting Temozolomide Loaded in Folate-Conjugated Magnetic Triblock Copolymer Nanoparticles to Improve the Therapeutic Efficiency of Rat Brain Gliomas. ACS Biomater. Sci. Eng..

[12] Waghule T., Rapalli V.K., Singhvi G., Gorantla S., Khosa A., Dubey S.K., Saha R.N. (2021). Design of temozolomide-loaded proliposomes and lipid crystal nanoparticles with industrial feasible approaches: Comparative assessment of drug loading, entrapment efficiency, and stability at plasma pH. J. Liposome Res..

[13] Song S., Mao G., Du J., Zhu X. (2016). Novel RGD containing, temozolomide-loading nanostructured lipid carriers for glioblastoma multiforme chemotherapy. Drug Deliv..

[14] Guo P., Moses-Gardner A., Huang J., Smith E.R., Moses M.A. (2019). ITGA2 as a potential nanotherapeutic target for glioblastoma. Sci. Rep..

[15] Alphandéry E. (2020). Nano-therapies for glioblastoma treatment. Cancers.

[16] Yasaswi P.S., Shetty K., Yadav K.S. (2021). Temozolomide nano enabled medicine: Promises made by the nanocarriers in glioblastoma therapy. J. Control. Release.

[17] Zhang C., Wu J., Liu W., Zheng X., Zhang W., Lee C.S., Wang P. (2020). Hypocrellin-Based Multifunctional Phototheranostic Agent for NIR-Triggered Targeted Chemo/Photodynamic/Photothermal Synergistic Therapy against Glioblastoma. ACS Appl. Bio Mater..

[18] Zhao M., van Straten D., Broekman M.L.D., Préat V., Schiffelers R.M. (2020). Nanocarrier-based drug combination therapy for glioblastoma. Theranostics.

[19] Di Filippo L.D., de Carvalho S.G., Duarte J.L., Luiz M.T., Paes Dutra J.A., de Paula G.A., Chorilli M., Conde J. (2023). A receptor-mediated landscape of druggable and targeted nanomaterials for gliomas. Mater. Today Bio.

[20] El-Sawy H.S., Al-Abd A.M., Ahmed T.A., El-Say K.M., Torchilin V.P. (2018). Stimuli-Responsive Nano-Architecture Drug-Delivery Systems to Solid Tumor Micromilieu: Past, Present, and Future Perspectives. ACS Nano.

[21] Kim A., Walz W., Agrahari V., Kim A., Agrahari V. (2021). Nanotherapy for Brain Tumor Drug Delivery.

[22] Krajcer A., Grzywna E., Lewandowska-Łańcucka J. (2023). Strategies increasing the effectiveness of temozolomide at various levels of anti-GBL therapy. Biomed. Pharmacother..

[23] Yang J., Xu Y., Fu Z., Chen J., Fan W., Wu X. (2023). Progress in research and development of temozolomide brain-targeted preparations: A review. J. Drug Target..

[24] Saraf S., Jain A., Tiwari A., Verma A., Panda P.K., Jain S.K. (2020). Advances in liposomal drug delivery to cancer: An overview. J. Drug Deliv. Sci. Technol..

[25] Guimarães D., Cavaco-Paulo A., Nogueira E. (2021). Design of liposomes as drug delivery system for therapeutic applications. Int. J. Pharm..

[26] Sheikholeslami B., Lam N.W., Dua K., Haghi M. (2022). Exploring the impact of physicochemical properties of liposomal formulations on their in vivo fate. Life Sci..

[27] Tiwari P., Yadav K., Shukla R.P., Gautam S., Marwaha D., Sharma M., Mishra P.R. (2023). Surface modification strategies in translocating nano-vesicles across different barriers and the role of bio-vesicles in improving anticancer therapy. J. Control. Release.

[28] Large D.E., Abdelmessih R.G., Fink E.A., Auguste D.T. (2021). Liposome composition in drug delivery design, synthesis, characterization, and clinical application. Adv. Drug Deliv. Rev..

[29] Kommineni N., Chaudhari R., Conde J., Tamburaci S., Cecen B., Chandra P., Prasad R. (2023). Engineered Liposomes in Interventional Theranostics of Solid Tumors. ACS Biomater. Sci. Eng..

[30] Moosavian S.A., Bianconi V., Pirro M., Sahebkar A. (2021). Challenges and pitfalls in the development of liposomal delivery systems for cancer therapy. Semin. Cancer Biol..

[31] Bulbake U., Doppalapudi S., Kommineni N., Khan W. (2017). Liposomal Formulations in Clinical Use: An Updated Review. Pharmaceutics.

[32] Amarandi R.-M., Ibanescu A., Carasevici E., Marin L., Dragoi B. (2022). Liposomal-Based Formulations: A Path from Basic Research to Temozolomide Delivery Inside Glioblastoma Tissue. Pharmaceutics.

[33] Vanza J., Jani P., Pandya N., Tandel H. (2018). Formulation and statistical optimization of intravenous temozolomide-loaded PEGylated liposomes to treat glioblastoma multiforme by three-level factorial design. Drug Dev. Ind. Pharm..

[34] Buzyurova D.N., Pashirova T.N., Zueva I.V., Burilova E.A., Shaihutdinova Z.M., Rizvanov I.K., Babaev V.M., Petrov K.A., Souto E.B. (2020). Surface modification of pralidoxime chloride-loaded solid lipid nanoparticles for enhanced brain reactivation of organophosphorus-inhibited AChE: Pharmacokinetics in rat. Toxicology.

[35] Quinn J.A., Jiang S.X., Reardon D.A., Desjardins A., Vredenburgh J.J., Rich J.N., Gururangan S., Friedman A.H., Bigner D.D., Sampson J.H. (2009). Phase II Trial of Temozolomide Plus O 6 -Benzylguanine in Adults With Recurrent, Temozolomide-Resistant Malignant Glioma. J. Clin. Oncol..

[36] Campolo M., Lanza M., Casili G., Paterniti I., Filippone A., Caffo M., Cardali S.M., Puliafito I., Colarossi C., Raciti G. (2020). TAK1 Inhibitor Enhances the Therapeutic Treatment for Glioblastoma. Cancers.

[37] Goker Bagca B., Ozates N.P., Asik A., Caglar H.O., Gunduz C., Biray Avci C. (2020). Temozolomide treatment combined with AZD3463 shows synergistic effect in glioblastoma cells. Biochem. Biophys. Res. Commun..

[38] Narayan R.S., Molenaar P., Teng J., Cornelissen F.M.G., Roelofs I., Menezes R., Dik R., Lagerweij T., Broersma Y., Petersen N. (2020). A cancer drug atlas enables synergistic targeting of independent drug vulnerabilities. Nat. Commun..

[39] Pereira I., Severino P., Santos A.C., Silva A.M., Souto E.B. (2018). Linalool bioactive properties and potential applicability in drug delivery systems. Colloids Surf. B Biointerfaces.

[40] Vengoji R., Macha M.A., Batra S.K., Shonka N.A. (2018). Natural products: A hope for glioblastoma patients. Oncotarget.

[41] Agarwal S., Muniyandi P., Maekawa T., Kumar D.S. (2018). Vesicular systems employing natural substances as promising drug candidates for MMP inhibition in glioblastoma: A nanotechnological approach. Int. J. Pharm..

[42] Carbone C., Martins-Gomes C., Caddeo C., Silva A.M., Musumeci T., Pignatello R., Puglisi G., Souto E.B. (2018). Mediterranean essential oils as precious matrix components and active ingredients of lipid nanoparticles. Int. J. Pharm..

[43] Crooker K., Aliani R., Ananth M., Arnold L., Anant S., Thomas S.M. (2018). A review of promising natural chemopreventive agents for head and neck cancer. Cancer Prev. Res..

[44] Zhang Q.-Y., Wang F.-X., Jia K.-K., Kong L.-D. (2018). Natural Product Interventions for Chemotherapy and Radiotherapy-Induced Side Effects. Front. Pharmacol..

[45] Limonta P., Moretti R., Marzagalli M., Fontana F., Raimondi M., Montagnani Marelli M. (2019). Role of Endoplasmic Reticulum Stress in the Anticancer Activity of Natural Compounds. Int. J. Mol. Sci..

[46] Dutta S., Mahalanobish S., Saha S., Ghosh S., Sil P.C. (2019). Natural products: An upcoming therapeutic approach to cancer. Food Chem. Toxicol..

[47] Liu K., Zhao F., Yan J., Xia Z., Jiang D., Ma P. (2020). Hispidulin: A promising flavonoid with diverse anti-cancer properties. Life Sci..

[48] Gomez-Cadena A., Barreto A., Fioretino S., Jandus C. (2020). Immune system activation by natural products and complex fractions: A network pharmacology approach in cancer treatment. Cell Stress.

[49] Domínguez-Martín E.M., Magalhães M., Efferth T., Díaz-Lanza A.M., Cabral C., Rijo P. (2023). Terpenes: A hope for glioblastoma patients. New Insights into Glioblastoma.

[50] Chang K.-F., Huang X.-F., Chang J.T., Huang Y.-C., Weng J.-C., Tsai N.-M. (2020). Cedrol suppresses glioblastoma progression by triggering DNA damage and blocking nuclear translocation of the androgen receptor. Cancer Lett..

[51] Chang K.-F., Huang X.-F., Chang J.T., Huang Y.-C., Lo W.-S., Hsiao C.-Y., Tsai N.-M. (2020). Cedrol, a Sesquiterpene Alcohol, Enhances the Anticancer Efficacy of Temozolomide in Attenuating Drug Resistance via Regulation of the DNA Damage Response and MGMT Expression. J. Nat. Prod..

[52] Hu J., Wang J., Wang G., Yao Z., Dang X. (2016). Pharmacokinetics and antitumor efficacy of DSPE-PEG2000 polymeric liposomes loaded with quercetin and temozolomide: Analysis of their effectiveness in enhancing the chemosensitization of drug-resistant glioma cells. Int. J. Mol. Med..

[53] Tsepaeva O.V., Nemtarev A.V., Abdullin T.I., Grigor’Eva L.R., Kuznetsova E.V., Akhmadishina R.A., Ziganshina L.E., Cong H.H., Mironov V.F. (2017). Design, Synthesis, and Cancer Cell Growth Inhibitory Activity of Triphenylphosphonium Derivatives of the Triterpenoid Betulin. J. Nat. Prod..

[54] Tsepaeva O.V., Salikhova T.I., Ishkaeva R.A., Kundina A.V., Abdullin T.I., Laikov A.V., Tikhomirova M.V., Idrisova L.R., Nemtarev A.V., Mironov V.F. (2023). Bifunctionalized Betulinic Acid Conjugates with C-3-Monodesmoside and C-28-Triphenylphosphonium Moieties with Increased Cancer Cell Targetability. J. Nat. Prod..

[55] Yang X.W., Li S.M., Shen Y.H., Zhang W.D. (2008). Phytochemical and biological studies of Abies species. Chem. Biodivers..

[56] Wu W., Chen X., Liu Y., Wang Y., Tian T., Zhao X., Li J., Ruan H. (2016). Triterpenoids from the branch and leaf of Abies fargesii. Phytochemistry.

[57] Babaie S., Del Bakhshayesh A.R., Ha J.W., Hamishehkar H., Kim K.H. (2020). Invasome: A Novel Nanocarrier for Transdermal Drug Delivery. Nanomaterials.

[58] Teaima M.H., Eltabeeb M.A., El-Nabarawi M.A., Abdellatif M.M. (2022). Utilization of propranolol hydrochloride mucoadhesive invasomes as a locally acting contraceptive: In-vitro, ex-vivo, and in-vivo evaluation. Drug Deliv..

[59] Song H., Wei M., Zhang N., Li H., Tan X.C., Zhang Y.J., Zheng W.S. (2018). Enhanced permeability of blood-brain barrier and targeting function of brain via borneol-modified chemically solid lipid nanoparticle. Int. J. Nanomed..

[60] Sinyashin K.O., Zakharova L.Y., Pashirova T.N., Petrov K.A., Voloshina A.D., Vyshtakalyuk A.B., RizvanovI K.H., Babaev V.M., Maganova F.I. (2021). Liposomal Form of Abies sibirica Resin for Non-Invasive Delivery of Biologically Active Substance of Abies sibirica Oleoresin to Brain, Which Has Antioxidant, Cytotoxic Activity against Cervical Cancer Cells, and Methods for Its Preparation.

[61] Khan A., Imam S.S., Aqil M., Ahad A., Sultana Y., Ali A., Khan K. (2016). Brain Targeting of Temozolomide via the Intranasal Route Using Lipid-Based Nanoparticles: Brain Pharmacokinetic and Scintigraphic Analyses. Mol. Pharm..

[62] Gelsleichter N.E., de Souza P.O., Teixeira F.C., Debom G.N., Lenz G.S., Roliano G.G., de Cássia Sant’ana R., Visioli F., Fachel F.N.S., Michels L.R. (2023). Metastatic Melanoma: A Preclinical Model Standardization and Development of a Chitosan-Coated Nanoemulsion Containing Temozolomide to Treat Brain Metastasis. Cell. Mol. Neurobiol..

[63] Hsieh Y.-S., Yang S.-F., Hsieh Y.-H., Hung C.-H., Chu S.-C., Yang S.-H., Chen P.-N. (2017). Erratum: “The Inhibitory Effect of Abietic Acid on Melanoma Cancer Metastasis and Invasiveness In Vitro and In Vivo”. Am. J. Chin. Med..

[64] Liu X., Chen W., Liu Q., Dai J. (2019). Abietic acid suppresses non-small-cell lung cancer cell growth via blocking IKKβ/NF-κB signaling. OncoTargets Ther..

[65] Voloshina A.D., Semenov V.E., Strobykina A.S., Kulik N.V., Krylova E.S., Zobov V.V., Reznik V.S. (2017). Synthesis and antimicrobial and toxic properties of novel 1,3-bis(alkyl)-6-methyluracil derivatives containing 1,2,3- and 1,2,4-triazolium fragments. Russ. J. Bioorganic Chem..

[66] Kirby C., Gregoriadis G. (1984). Dehydration-Rehydration Vesicles: A Simple Method for High Yield Drug Entrapment in Liposomes. Nat. Biotechnol..

[67] Pashirova T.N., Sapunova A.S., Lukashenko S.S., Burilova E.A., Lubina A.P., Shaihutdinova Z.M., Gerasimova T.P., Kovalenko V.I., Voloshina A.D., Souto E.B. (2020). Synthesis, structure-activity relationship and biological evaluation of tetracationic gemini Dabco-surfactants for transdermal liposomal formulations. Int. J. Pharm..

[68] Hsieh Y.S., Yang S.F., Hsieh Y.H., Hung C.H., Chu S.C., Yang S.H., Chen P.N. (2015). The Inhibitory Effect of Abietic Acid on Melanoma Cancer Metastasis and Invasiveness In Vitro and In Vivo. Am. J. Chin. Med..

[69] Terstappen G.C., Meyer A.H., Bell R.D., Zhang W. (2021). Strategies for delivering therapeutics across the blood–brain barrier. Nat. Rev. Drug Discov..

[70] Costa C., Moreira J.N., Amaral M.H., Sousa Lobo J.M., Silva A.C. (2019). Nose-to-brain delivery of lipid-based nanosystems for epileptic seizures and anxiety crisis. J. Control. Release.

[71] Chandler S.G., Ilium L., Thomas N.W. (1991). Nasal absorption in rats. II. Effect of enhancers on insulin absorption and nasal histology. Int. J. Pharm..

[72] Williams A.C., Barry B.W. (2004). Penetration enhancers. Adv. Drug Deliv. Rev..

[73] Lu Y., Du S., Bai J., Li P., Wen R., Zhao X. (2012). Bioavailability and Brain-Targeting of Geniposide in Gardenia-Borneol Co-Compound by Different Administration Routes in Mice. Int. J. Mol. Sci..

[74] Wang L., Zhao X., Du J., Liu M., Feng J., Hu K. (2019). Improved brain delivery of pueraria flavones via intranasal administration of borneol-modified solid lipid nanoparticles. Nanomedicine.

[75] Kulkarni M., Sawant N., Kolapkar A., Huprikar A., Desai N. (2021). Borneol: A Promising Monoterpenoid in Enhancing Drug Delivery Across Various Physiological Barriers. AAPS PharmSciTech.

[76] Wang L., Xu L., Du J., Zhao X., Liu M., Feng J., Hu K. (2021). Nose-to-brain delivery of borneol modified tanshinone IIA nanoparticles in prevention of cerebral ischemia/reperfusion injury. Drug Deliv..

[77] Suckling K.E., Blair H.A.F., Boyd G.S., Craig I.F., Malcolm B.R. (1979). The importance of the phospholipid bilayer and the length of the cholesterol molecule in membrane structure. Biochim. Biophys. Acta Biomembr..

[78] Owensiii D., Peppas N. (2006). Opsonization, biodistribution, and pharmacokinetics of polymeric nanoparticles. Int. J. Pharm..

[79] Zhang X., Wang H., Ma Z., Wu B. (2014). Effects of pharmaceutical PEGylation on drug metabolism and its clinical concerns. Expert Opin. Drug Metab. Toxicol..

[80] Kolate A., Baradia D., Patil S., Vhora I., Kore G., Misra A. (2014). PEG—A versatile conjugating ligand for drugs and drug delivery systems. J. Control. Release.

[81] Aranda F.J., Villalaín J. (1997). The interaction of abietic acid with phospholipid membranes. Biochim. Biophys. Acta Biomembr..

[82] Villalaín J. (1997). Location of the toxic molecule abietic acid in model membranes by MAS–NMR. Biochim. Biophys. Acta Biomembr..

[83] Lin C.-H., Chuang H.-S. (2004). Use of abietic acid or derivative thereof for modulation permeability of plasma membrane.

[84] Faustino C., Neto Í., Fonte P., Macedo A. (2019). Cytotoxicity and Chemotherapeutic Potential of Natural Rosin Abietane Diterpenoids and their Synthetic Derivatives. Curr. Pharm. Des..

[85] Zentar H., Jannus F., Gutierrez P., Medina-O’Donnell M., Lupiáñez J.A., Reyes-Zurita F.J., Alvarez-Manzaneda E., Chahboun R. (2022). Semisynthesis and Evaluation of Anti-Inflammatory Activity of the Cassane-Type Diterpenoid Taepeenin F and of Some Synthetic Intermediates. J. Nat. Prod..

[86] Haffez H., Osman S., Ebrahim H.Y., Hassan Z.A. (2022). Growth Inhibition and Apoptotic Effect of Pine Extract and Abietic Acid on MCF-7 Breast Cancer Cells via Alteration of Multiple Gene Expressions Using In Vitro Approach. Molecules.

[87] Kawaoka Y., Feng H., Watanabe T., Yamashita M. (2021). Adjuvant and vaccine containing adjuvant.

[88] Xu Y., Tong Y., Lei Z., Zhu J., Wan L. (2023). Abietic acid induces ferroptosis via the activation of the HO-1 pathway in bladder cancer cells. Biomed. Pharmacother..

