# Supplementary material for: Terpenes-Modified Lipid Nanosystems for Temozolomide, Improving Cytotoxicity against Glioblastoma Human Cancer Cells In Vitro
Source: Nanomaterials (Basel). 2023 Dec 24;14(1):55. doi: 10.3390/nano14010055 (PMC10780480; doi:10.3390/nano14010055)
Supplement: Supplementary file 1 [file nanomaterials-14-00055-s001.zip › nanomaterials-2663913-supplementary.pdf]

nanomaterials

Supplementary Materials

Table S1. Identified components of *Abies sibirica* resin.

№	Compound	Time (min)	Formula	Molecular weight	Content (%)
1	Tricyclene	2.29[a]	$C_{10}H_{16}$	136	0.6
2	α-Pinene	2.41[a]	$C_{10}H_{16}$	136	9.2
3	Camphene	2.59[a]	$C_{10}H_{16}$	136	5.8
4	β-Pinene	2.93[a]	$C_{10}H_{16}$	136	3.6
5	β-Myrcene	3.35[a] 5.24[b]	$C_{10}H_{16}$	136	1.1
6	Limonene	3.61[a] 5.81[b]	$C_{10}H_{16}$	136	4.0
7	Borneol	5.64[a]	$C_{10}H_{18}O$	154	1.2
8	Bornyl acetate	7.33[a] 15.50[b]	$C_{12}H_{20}O_2$	196	9.6
9	Caryophyllene	9.13[a] 20.56[b]	$C_{15}H_{24}$	204	2.8
10	Humulene	9.57[a] 21.88[b]	$C_{15}H_{24}$	204	1.3
11	Borneol-TMS[c]	12.99[b]	$C_{13}H_{26}OSi$	226	–
12	α-Bisabolol-TMS[c]	33.39[b]	$C_{18}H_{34}OSi$	294	2.2
13	Manool-TMS[c]	45.90[b]	$C_{23}H_{42}OSi$	363	7.7
14	Diterpene alcohol-TMS[c]	46.99[b]	$C_{23}H_{42}OSi$	363	8.4
15	Pimaric acid-TMS[c]	50.37[b]	$C_{23}H_{38}O_2Si$	374	0.8
16	Isopimaric acid-TMS[c]	50.68[b]	$C_{23}H_{38}O_2Si$	374	3.6
15	Abietic acid isomer-TMS[c]	51.43[b]	$C_{23}H_{38}O_2Si$	374	3.5
16	Abietic acid isomer-TMS[c]	51.86[b]	$C_{23}H_{38}O_2Si$	374	4.1
17	Abietic acid-TMS[c]	53.22[b]	$C_{23}H_{38}O_2Si$	374	17.8
18	Abietic acid isomer-TMS[c]	55.35[b]	$C_{23}H_{38}O_2Si$	374	9.1

[a] The times are from chromatogram obtained by Method A; [b] The times are from chromatogram obtained by Method B; [c] Trimethylsilylated derivative.

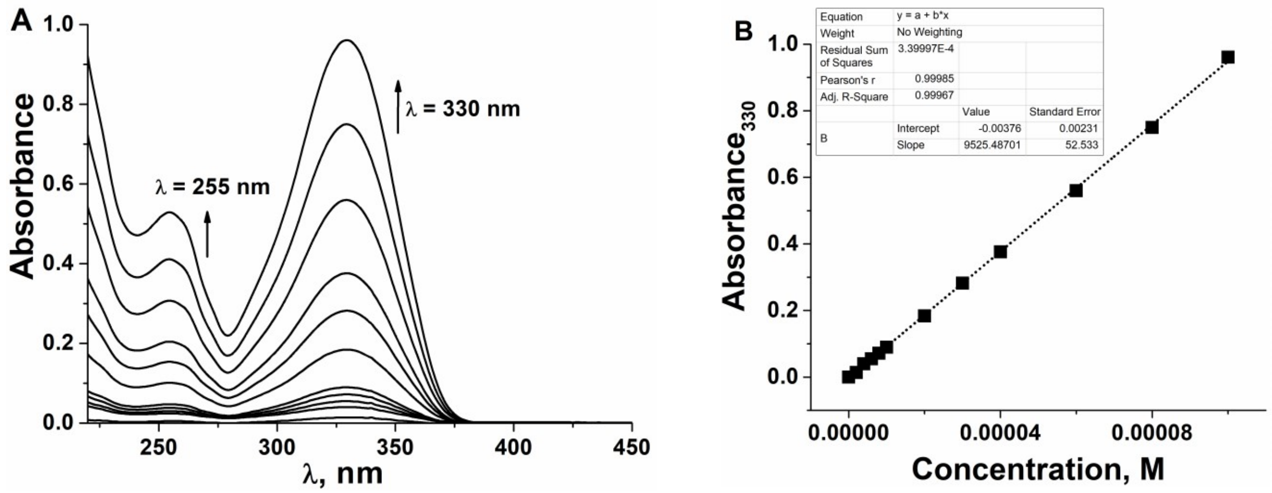

Figure S1. Spectra (**A**) and calibration curve at 330 nm (**B**) of TMZ in acetate buffer, pH = 4, L=1 cm, 25 °C.

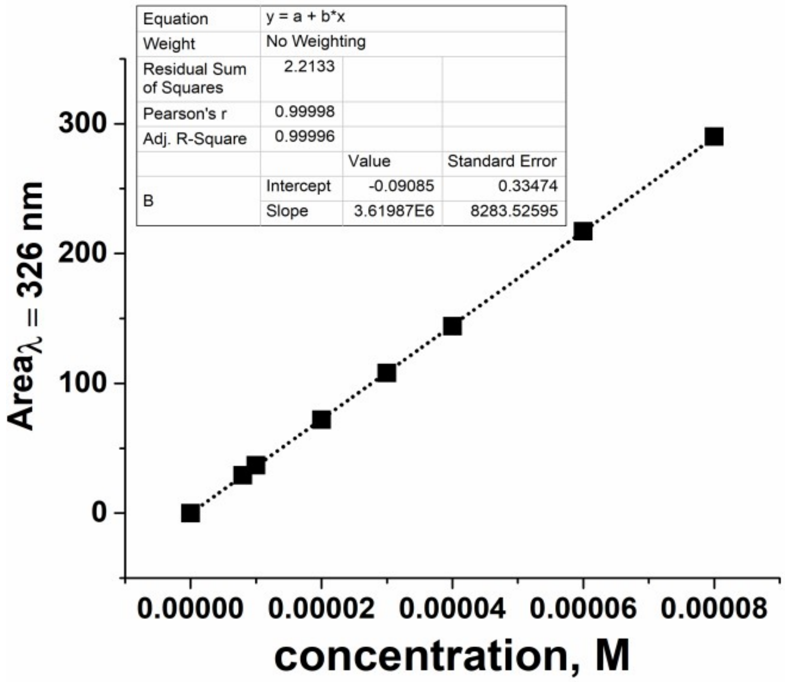

Figure S2. Calibration curve at 326 nm of TMZ in acetate buffer using an HPLC detection method, pH = 4, 25 °C.

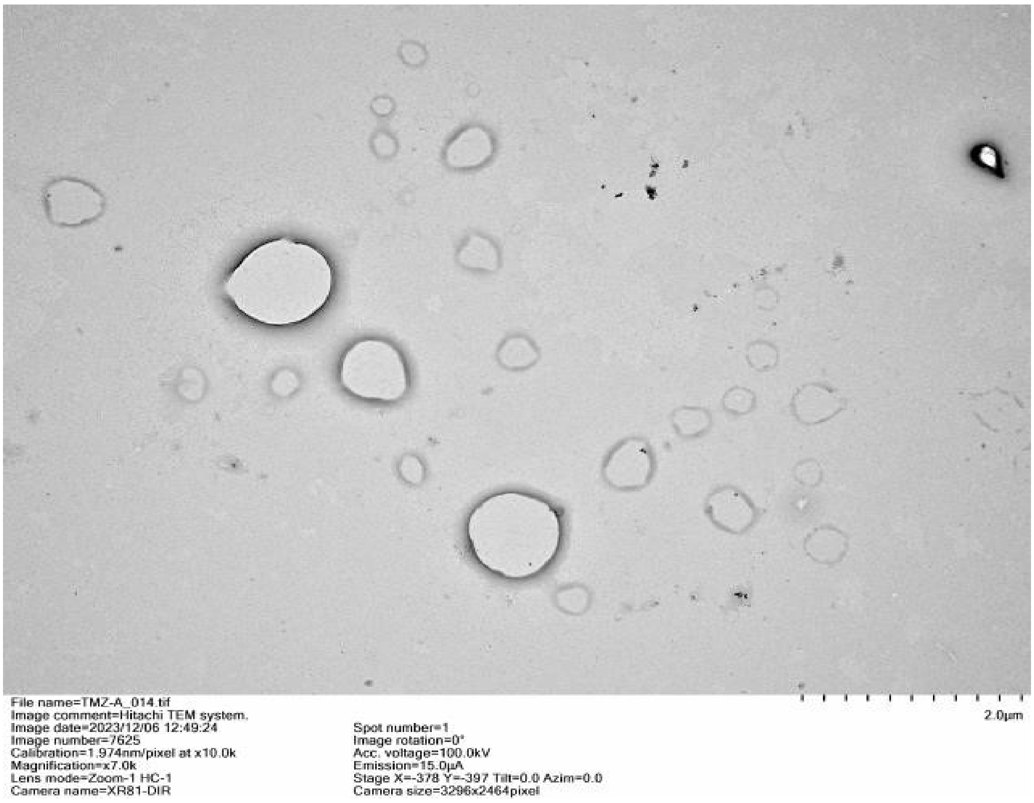

(**a**)

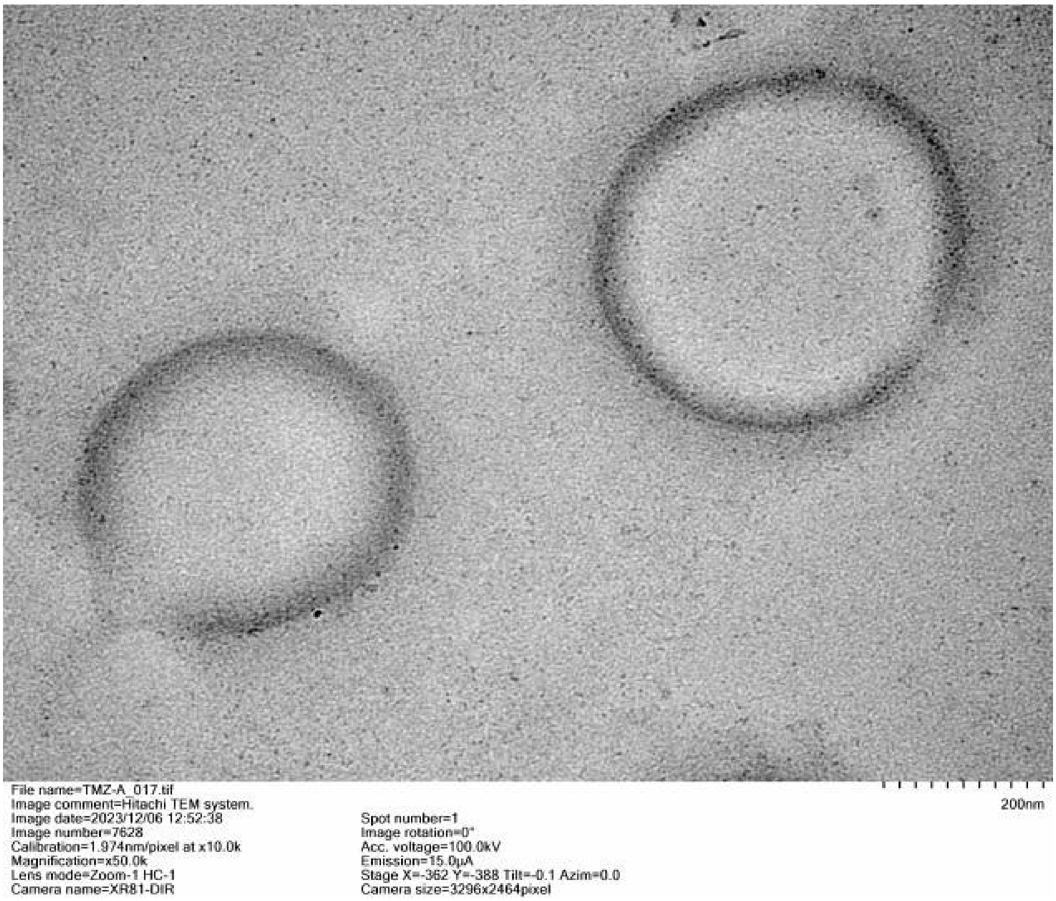

(**b**

Figure S3. TEM imaging of TMZ-loaded abietic acid–PC/DSPE–PEG_{2000}/Ch lipid nanosystems, C_{PC} = 0.58 µg/mL, mQ water, 25 °C.

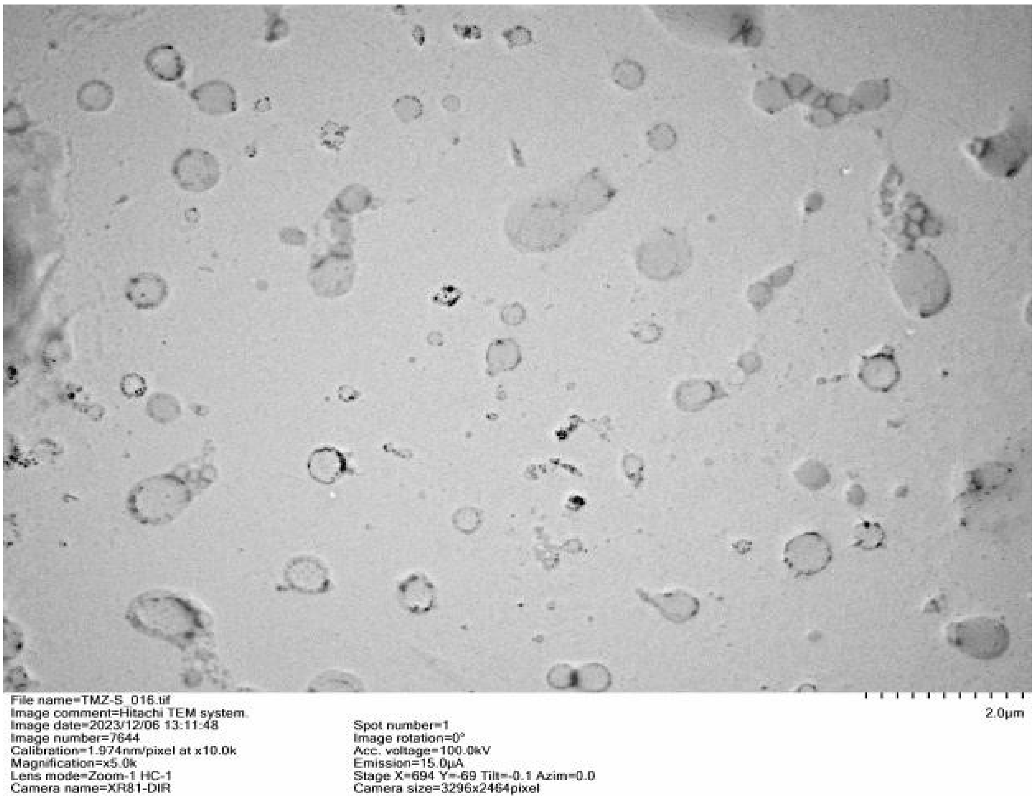

(**a**)

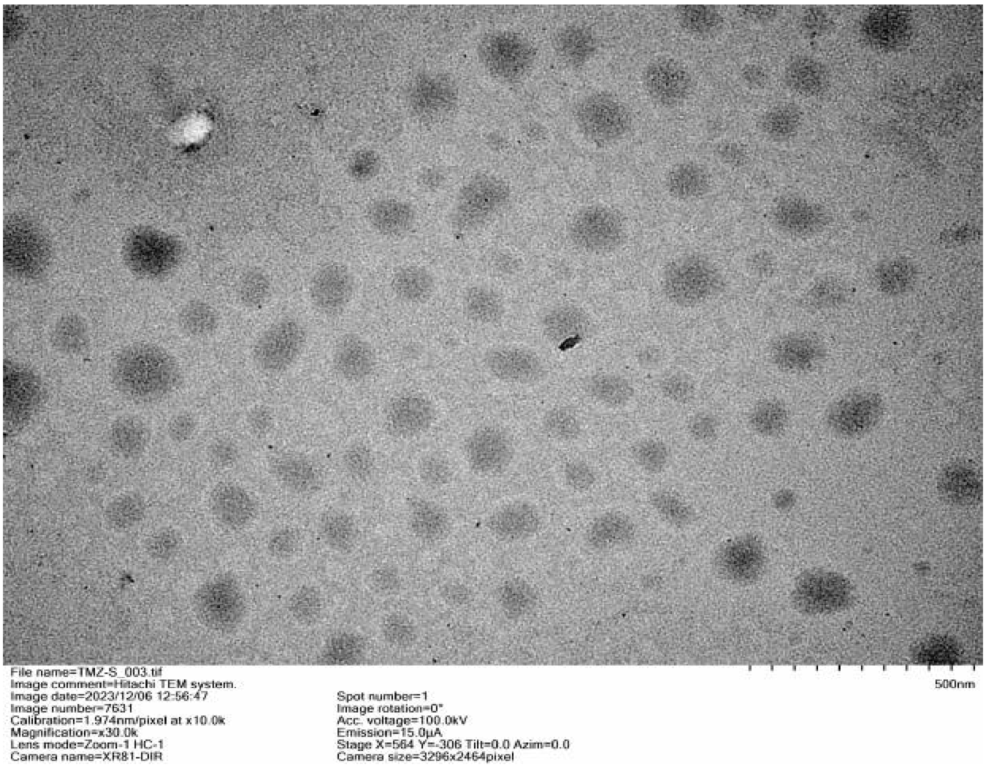

(**c**)

Figure S4. TEM imaging of TMZ-loaded *Abies sibirica*–PC/DSPE–PEG_{2000}/Ch lipid nanosystems, C_{PC} = 0.58 μg/mL, mQ water, 25 °C.

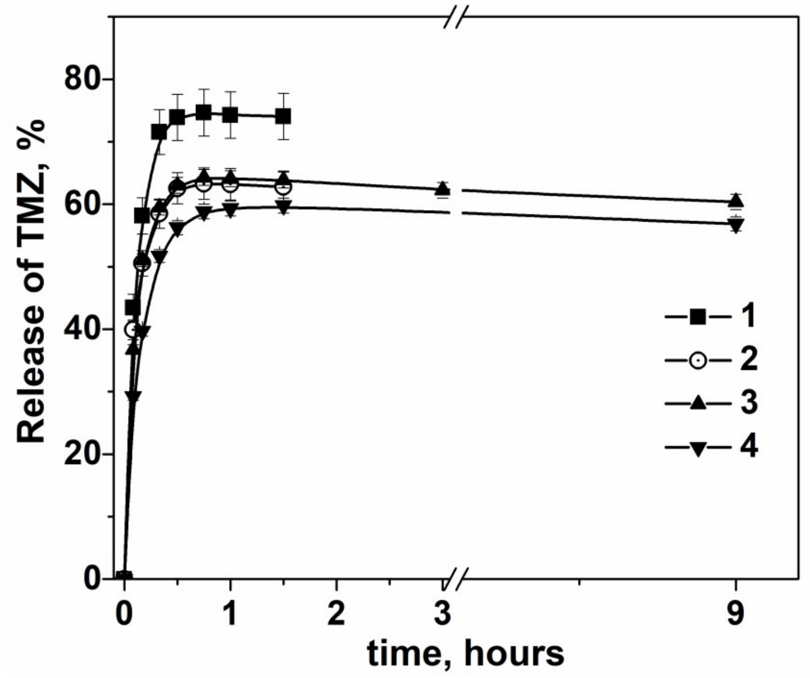

Figure S5. In vitro TMZ release from buffer solution (1), lipid nanosystem (2), abietic acid–lipid system (3), *A. sibirica*–lipid nanosystem (4) using the dialysis bag method (n=3, experiments were replicated in triplicate) and HPLC detection, acetate buffer (0.01 M), pH=4, 37 °C.

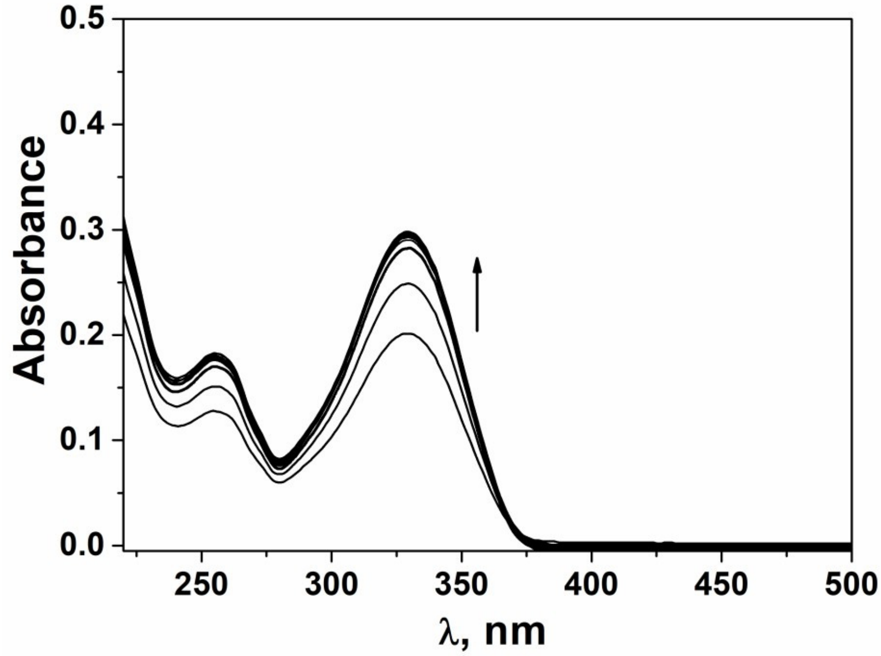

Figure S6. Spectra of TMZ in acetate buffer (0.01 M), recorded during release time, free solution, pH=4, L=1 cm, 25 °C.

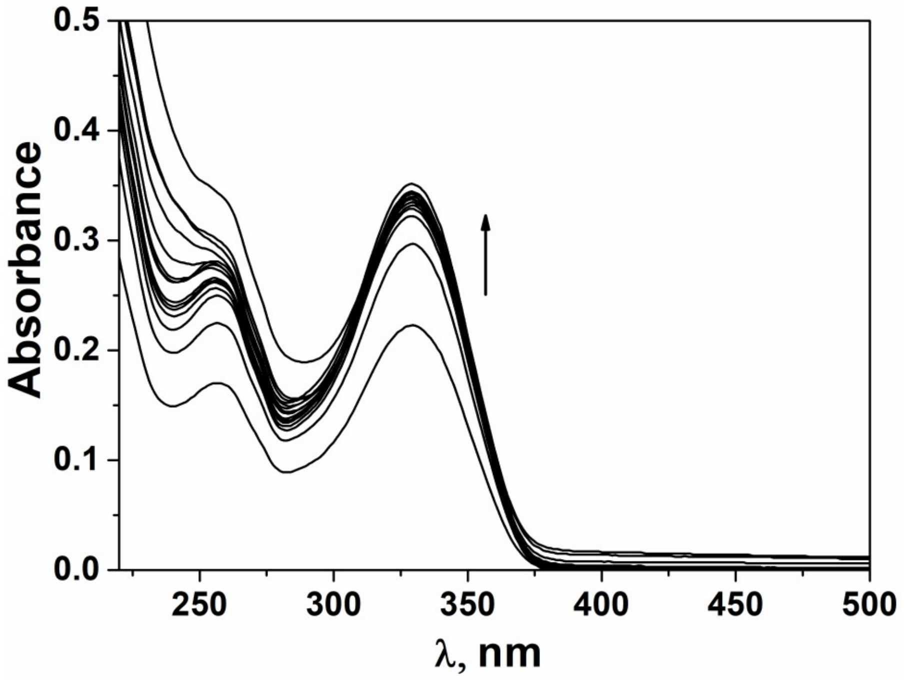

Figure S7. Spectra of TMZ in acetate buffer (0.01 M), recorded during release time from TMZ-loaded lipid system, pH=4, L=1 cm, 25 °C.

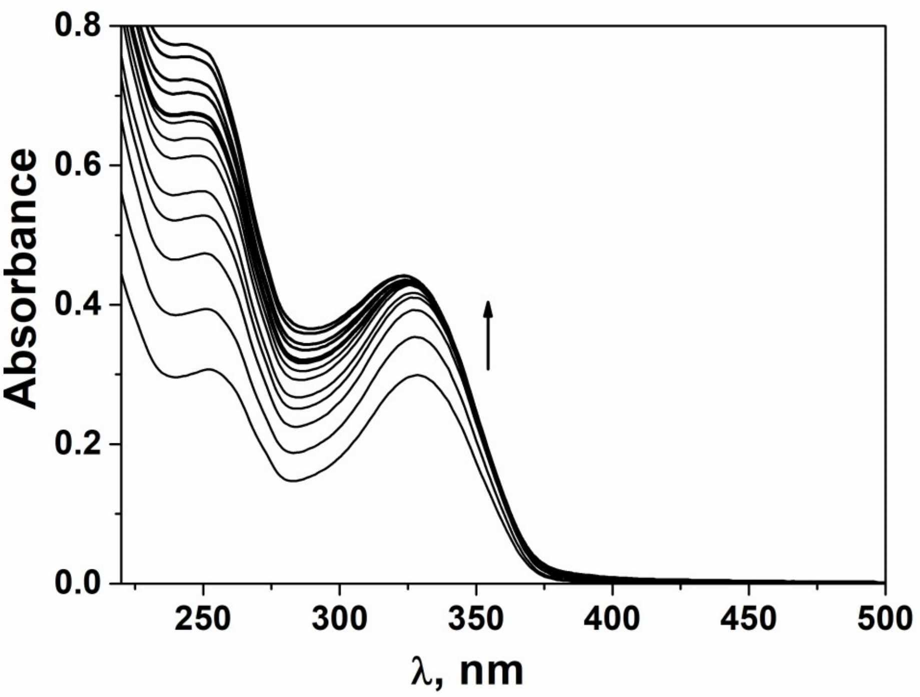

Figure S8. Spectra of TMZ in acetate buffer (0.01 M), recorded during release time from TMZ-loaded–abietic acid–lipid system, pH=4, L=1 cm, 25 °C.

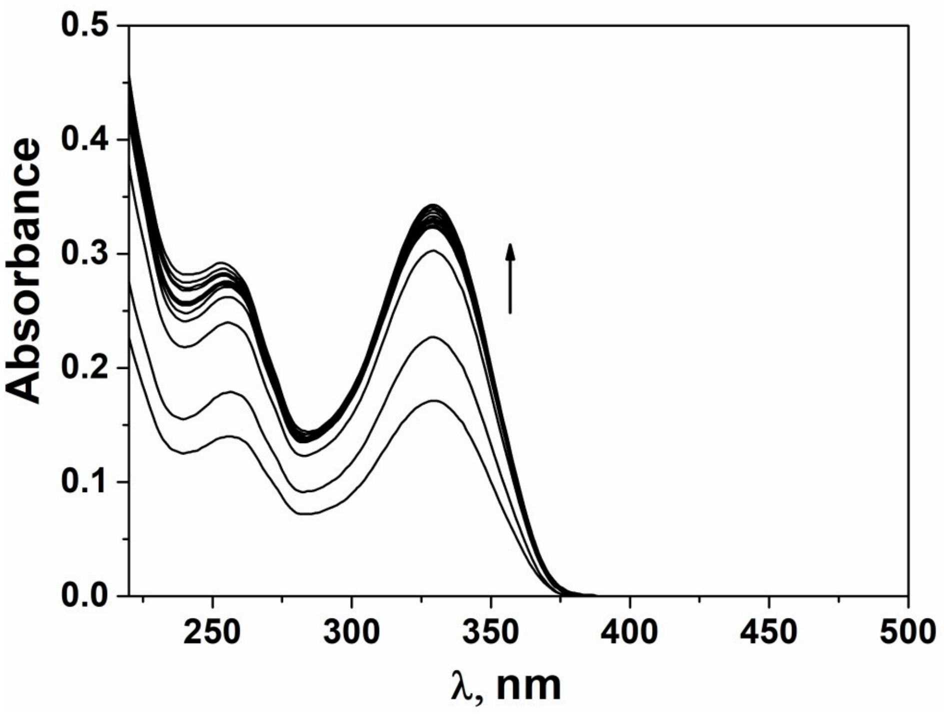

Figure S9. Spectra of TMZ in acetate buffer (0.01 M), recorded during release time from TMZ-loaded–*Abies sibirica*–lipid system, pH=4, L=1 cm, 25 °C.
